# Endovascular Device Choice and Tools for Recanalization of Medium Vessel Occlusions: Insights From the MeVO FRONTIERS International Survey

**DOI:** 10.3389/fneur.2021.735899

**Published:** 2021-09-15

**Authors:** Nima Kashani, Petra Cimflova, Johanna M. Ospel, Nishita Singh, Mohammed A. Almekhlafi, Jeremy Rempel, Jens Fiehler, Michael Chen, Nobuyuki Sakai, Ronit Agid, Manraj Heran, Manon Kappelhof, Mayank Goyal

**Affiliations:** ^1^Department of Diagnostic Imaging, Foothills Medical Center, University of Calgary, Calgary, AB, Canada; ^2^Department of Clinical Neurosciences, Foothills Medical Center, University of Calgary, Calgary, AB, Canada; ^3^Department of Medical Imaging, St. Anne's University Hospital Brno and Faculty of Medicine, Masaryk University, Brno, Czech Republic; ^4^Department of Radiology, University Hospital of Basel, Basel, Switzerland; ^5^Department of Diagnostic Imaging, University of Alberta Hospital, University of Alberta, Edmonton, AB, Canada; ^6^Department of Neuroradiology, University Medical Center Hamburg-Eppendorf, Hamburg, Germany; ^7^Department of Neurological Sciences, Rush University Medical Center, Chicago, IL, United States; ^8^Department of Neurosurgery, Kobe City Medical Center General Hospital, Kobe, Japan; ^9^Department of Neuroradiology, Toronto Western Hospital, Toronto, ON, Canada; ^10^Department of Neuroradiology, Vancouver General Hospital, Toronto, ON, Canada; ^11^Department of Radiology and Nuclear Medicine, Amsterdam University Medical Centers, University of Amsterdam, Amsterdam, Netherlands

**Keywords:** acute ischemic stroke, endovascular thrombectomy, aspiration thrombectomy, medium vessel occlusions, endovascular treatment (EVT), MeVO, stroke, neurointervention

## Abstract

**Background:** Endovascular treatment (EVT) for stroke due to medium vessel occlusion (MeVO) can be technically challenging. Devices and tools are rapidly evolving. We aimed to gain insight into preferences and global perspectives on the usage of endovascular tools in treating MeVOs.

**Methods:** We conducted an international survey with seven scenarios of patients presenting A3, M2/3, M3, M3/4, or P2/3 occlusions. Respondents were asked for their preferred first-line endovascular approach, and whether they felt that the appropriate endovascular tools were available to them. Answers were analyzed by occlusion location and geographical region of practice, using multinomial/binary logistic regression.

**Results:** A total of 263 neurointerventionists provided 1836 responses. The first-line preferences of physicians were evenly distributed among stent-retrievers, combined approaches, and aspiration only (33.2, 29.8, and 26.8%, respectively). A3 occlusions were more often treated with stent-retrievers (RR 1.21, 95% CI: 1.07–1.36), while intra-arterial thrombolysis was more often preferred in M3 (RR 2.47, 95% CI: 1.53–3.98) and M3/4 occlusions (RR 7.71, 95% CI: 4.16–14.28) compared to M2/3 occlusions. Respondents who thought appropriate tools are currently not available more often chose stent retrievers alone (RR 2.07; 95% CI: 1.01–4.24) or intra-arterial thrombolysis (RR 3.35, 95% CI: 1.26–8.42). Physicians who stated that they do not have access to optimal tools opted more often not to treat at all (RR 3.41, 95% CI: 1.11–10.49). Stent-retrievers alone were chosen more often and contact aspiration alone less often as a first-line approach in Europe (RR 2.12, 95% CI: 1.38–3.24; and RR 0.49, 95% CI 0.34–0.70, respectively) compared to the United States and Canada.

**Conclusions:** In EVT for MeVO strokes, neurointerventionalists choose a targeted vessel specific first-line approach depending on the occlusion location, region of practice, and availability of the appropriate tools.

## Introduction

Given the high efficacy of endovascular treatment (EVT) for acute ischemic stroke due to large vessel occlusion (LVO) and recently recognized substantial morbidity associated with stroke due to medium vessel occlusions (MeVO; distal M2/3, A2/3, P2/3 vessel segments) (1), EVT is now increasingly considered as a treatment for MeVO stroke (2), despite the lack of high-level evidence for MeVO EVT (3, 4). The smaller caliber, more distal location, and longer and more tortuous course of the affected vessels of MeVO compared to LVO makes EVT for MeVO stroke more challenging. Thinner, more fragile arterial walls could increase the risk of dissection, perforation, and vasospasm—complications that could offset any benefit of EVT (3, 4).

Currently, EVT tools and techniques are rapidly evolving, resulting in improved efficacy and safety of MeVO EVT. Several authors report promising results of primary aspiration as a first-line approach in MeVO stroke (5–9). Mini stent-retrievers are designed specifically for more distal occlusion locations, and novel approaches like the blind exchange mini-pinning (10, 11) technique may lead to higher rates of first-pass recanalization and a lower incidence of symptomatic intracranial hemorrhage compared to the use of mini stent retrievers alone (10).

However, in light of these developments, and in the absence of guideline-based treatment recommendations, clinical practice with regard to EVT techniques for MeVO stroke may vary greatly between countries or individual physicians. Currently, there are little data on the variability in MeVO EVT approaches. Therefore, we sought to determine global patterns in preferences and utilization of EVT devices in MeVO stroke. In addition, we explored interventionalists' access to the appropriate EVT devices, and whether they thought that appropriate tools already exist and are available to them in their current practice.

## Methods

We conducted an international, cross-sectional, anonymous, invitation-only survey: MeVO-FRONTIERS (MeVO-Finding Rationales and Objectifying New Targets for IntervEntional Revascularization in Stroke). Approximately 1,400 stroke physicians from 44 countries were invited to participate in this survey through Qualtrics (www.qualtrics.com). There were no restrictions for respondents based on country, years of experience, career stage, or hospital setting. The current study analyzes the survey questions on EVT technique and includes responses from interventionalists who identified themselves as neuroradiologists, neurosurgeons performing endovascular procedures, and interventional neurologists.

Response data were obtained from November 12, 2020 to December 31, 2020. Data are available from the corresponding author upon request. Approval by the local research ethics board of the University of Calgary was obtained (REB20-2086).

### Survey Design

The survey consisted of seven narrative MeVO cases with illustrative images and three to six clinical case vignettes per scenario. The case vignettes included patient demographics, clinical symptoms, radiological images, and imaging-derived information like CT-perfusion volumes or early ischemic changes on non-contrast CT. At the end of each case, physicians were asked what their preferred first-line EVT approach for that particular case would be. Answer options were (a) stent-retriever alone, (b) aspiration alone, (c) combined stent-retriever and aspiration, (d) intra-arterial thrombolysis, or (e) no treatment. Participants were then asked whether they thought that optimal tools for treating MeVOs with EVT currently exist (Yes/No/There is substantial scope for improvement) and whether the appropriate material for MeVO EVT is available in their institution (full survey questions shown in Supplementary Methods). Prior to accessing the case scenarios, physicians provided basic personal data (age group, gender, subspecialty, years of experience in stroke treatment, annual center stroke treatment volume, and geographic region).

### Statistical Analysis

Respondents' baseline characteristics were described using appropriate descriptive statistics. Univariable multinomial regression clustered by participant was used to assess the effect of occlusion site and respondent characteristics on preferred first-line EVT approach. Incidence rate ratios (IRRs) with 95% confidence intervals (CI) were reported.

For the following analysis, first-line approach was dichotomized into dummy variables (e.g., stent-retriever vs. others, aspiration vs. others, etc.). Then, preferred first-line EVT approach by occlusion site (M2/3, M3, M3/4, A3, P2/3) and region of practice (USA and Canada, Europe, rest of the world) were analyzed using binary logistic regression clustered by participant to calculate risk ratios (RRs) with 95% confidence intervals. M2/3 occlusion and responses from practitioners from the USA and Canada were chosen as reference values.

Multinomial regression analysis was used to determine treatment approach preference based on the existence and availability of specific endovascular tools and whether the interventionists thought they had adequate access to them in their current practice. In the multinomial regression model, the combined technique (stent retriever together with the contact aspiration) was chosen as the reference value and responses “Yes, tools exist” and “Yes, tools are available” were taken as base outcome.

The significance level was set at *p* < 0.05. Data analyses were performed in Stata 16.1 (StataCorp, College Station, TX, USA). Figures were created using the Microsoft PowerBI Platform.

## Results

In total, 263 of 366 stroke physicians who participated were neurointerventionists and were included in the current study. Seven clinical scenarios (two M2/3 and two A3 cases; one M3, M3/4, and P2/3 case) resulted in 1,836 responses on treatment approaches for MeVOs. Detailed respondent characteristics are listed in Supplementary Table 1.

Overall, physicians opted for first-line stent-retrievers alone in 33.2% (610/1,836) of cases, a combined approach in 29.8% (547/1,836), aspiration only in 26.8% (492/1,836) cases, and intra-arterial thrombolysis in 6.5% (119/1,836) cases. In 2.9% (54/1,836) of cases, no treatment was preferred, and in 0.8% (14/1,836) of responses, another endovascular approach was preferred (e.g., combined aspiration and stent-retriever with additional intra-arterial thrombolysis, or guidewire/microcatheter manipulation only). There were no differences in the preferred first-line approach among physicians based on their age, gender, years of experience in neurointervention, career stage, or annual institutional EVT volumes (Supplementary Table 2).

### First-Line Device Preference by Occlusion Site

**Figure 1:**
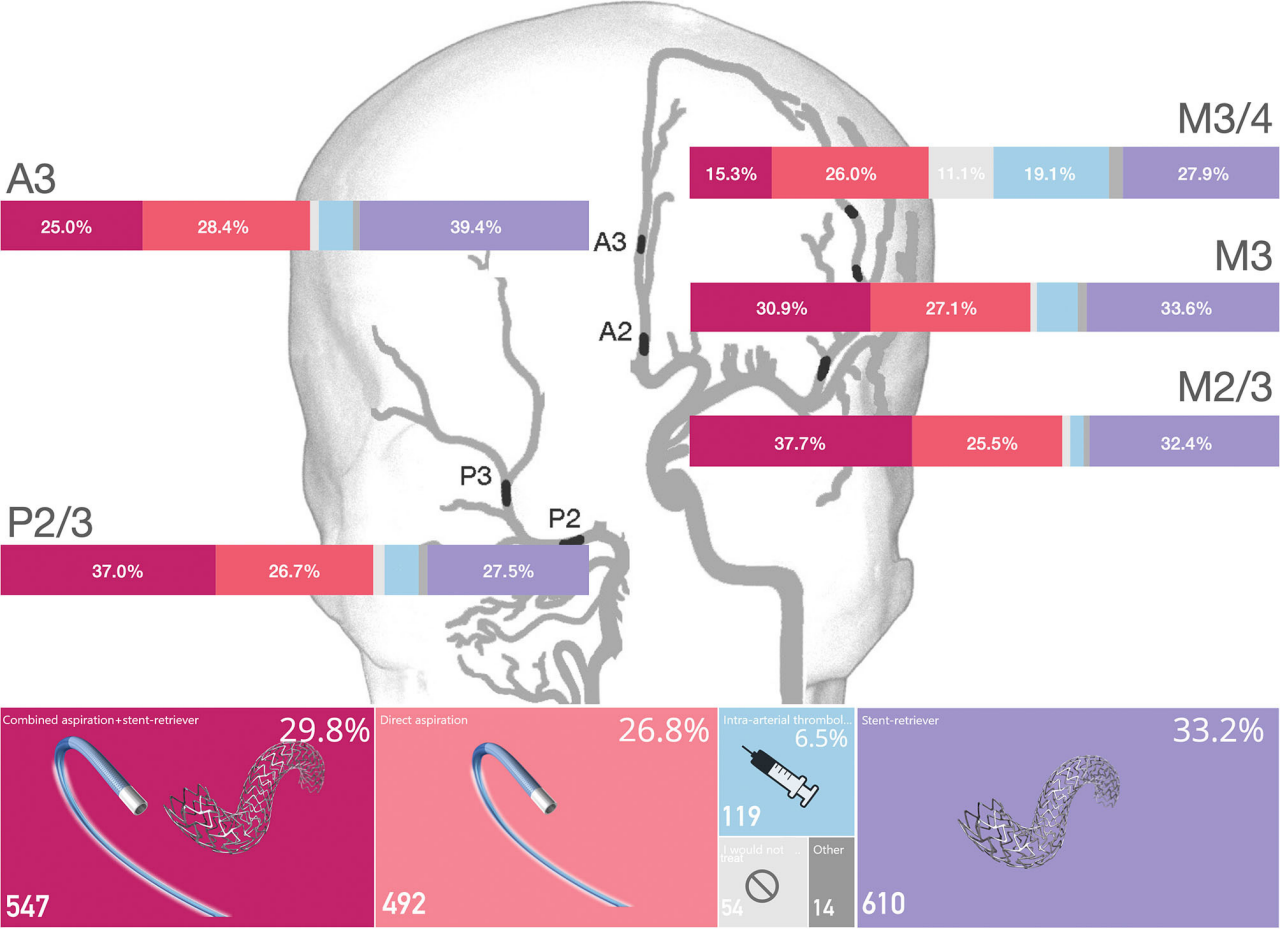
First-line treatment approach by MeVO occlusion site. Overall device usage and proportion of the respondents that chose each treatment approach in each specific vessel is shown in percentages.

First-line treatment approach for each occlusion site is shown in Figure 1 and Table 1. Preference for stent-retrievers alone did not differ between middle cerebral artery (MCA) occlusions (M2/3, M3, M3/4; Supplementary Table 2). In A3 occlusions, stent-retrievers were the most commonly preferred approach [39.4%, 207/525 responses; RR 1.22, 95% CI 1.08–1.37 (reference category: M2/3)]. For P2/3 occlusions, the combined approach (stent-retriever and aspiration) was frequently chosen (37% in P2/3). The combined technique was preferred less often in more distal MCA target occlusions (37.7% in M2/3 occlusions, 30.9% for M3, 15.3% for M3/4) with relative RRs indicating diminished use for very distal occlusions (Table 1). In more distal occlusions, respondents were also more likely to choose intra-arterial thrombolysis as a first-line approach (overall: RR 1.12; 95% CI 1.04–1.22) or to not treat with EVT at all (in M3/4: RR 8.30; 95% CI 3.54–19.48).

**Table 1:** Likelihood of preferred first-line approach (stent-retriever, combined stent-retriever and aspiration, aspiration, intra-arterial thrombolysis, or other) per occlusion site (reference: M2/3).

	**Overall (*n* = 1,836)**	**M3 (*n* = 262)**	**M3/4 (*n* = 262)**	**A3 (*n* = 525)**	**P2/3 (*n* = 262)**
	**RR (95% CI)**	**RR (95% CI)**	**RR (95% CI)**	**RR (95% CI)**	**RR (95% CI)**
First-line treatment approach for MeVO occlusion site compared to M2/3 occlusion^*^					
SR	1.01 (0.98–1.04)	1.04 (0.92–1.17)	0.86 (0.72–1.03)	**1.22 (1.08**–**1.37)**	**0.85 (0.73**–**0.99)**
Combined (SR + contact aspiration)	**0.93 (0.90**–**0.97)**	**0.82 (0.72**–**0.94)**	**0.40 (0.31**–**0.52)**	**0.66 (0.56**–**0.78)**	0.98 (0.86–1.12)
Contact aspiration	1.02 (0.98–1.06)	1.06 (0.92–1.22)	1.02 (0.86–1.20)	1.11 (0.97–1.27)	1.05 (0.90–1.22)
IAT	**1.12 (1.04**–**1.22)**	**2.47 (1.53**–**3.98)**	**7.71 (4.16**–**14.3)**	**1.92 (1.04**–**3.56)**	**2.31 (1.30**–**4.12)**
No treatment	1.10 (0.97–1.25)	0.86 (0.24–3.07)	**8.30 (3.54**–**19.5)**	1.43 (0.53–3.87)	1.43 (0.52–3.97)
Other^#^	1.07 (0.78–1.46)	2.00 (0.54–7.42)	1.34 (0.18–10.1)	1.00 (0.20–4.97)	2.00 (0.54–7.42)

*Risk ratios shown are from dichotomized comparisons, e.g., stent-retrievers vs. all other, contact aspiration vs. all other, etc. The “overall” column shows the risk ratio for preferring the row's first-line approach, for an increasingly distal occlusion location (from M2/3 to P2/3).*

* *M2/3 occlusion was chosen as reference value.*

# *“Other” category included free-text alternative answers, like combined stent-retriever, aspiration and intra-arterial thrombolysis, or intra-arterial thrombolysis and aspiration. 95% CI, 95% confidence interval; A3, third segment of anterior cerebral artery; IAT, intra-arterial thrombolysis; M2/3/4, second/third/fourth segments of middle cerebral artery; P2/3, second/third segment of posterior cerebral artery; RR, risk ratio; SR, stent-retriever. Bold values represent statistically significant findings (p < 0.05)*.

### Geographic Variations in Endovascular Device Choice

Stent-retriever alone was the most frequently chosen first-line approach in Europe [37.3%, 340/912 responses, RR 2.09, 95% CI 1.38–3.18 (reference category: USA and Canada)] and the rest of the world (43.3%, 179/413 responses, RR 2.43, 95% CI 1.57–3.78).

In the USA and Canada, contact aspiration alone was the most frequently preferred first-line approach (43.3%, 221/511 responses); in contrast to that, it was significantly less often chosen by Europeans [RR 0.49, 95% CI 0.34–0.70 (reference category: USA and Canada)] and practitioners from the rest of the world (RR 0.44, 95% CI 0.27–0.71).

Combined aspiration and stent-retrievers were chosen by European practitioners in 35.3% of cases (322/911 responses), which was significantly a more frequent choice when compared to the USA and Canada respondents (RR 1.41, 95% CI 1.10–1.96). There was no difference in choice of combined aspiration and stent-retriever between interventionalists in the USA and Canada and the rest of the world (25.1%, 128/511 vs. 23.5%, 97/413, respectively). There was no significant difference in the preference of intra-arterial thrombolysis or other endovascular techniques based on the region of practice (Table 2).

**Table 2:** Likelihood of preferred first-line endovascular treatment approach across world regions (reference: USA and Canada).

	**Overall (*n* = 1,836)**	**Europe (*n* = 912)**	**Rest of the world (*n* = 413)**
	**RR (95% CI)**	**RR (95% CI)**	**RR (95% CI)**
Endovascular technique choice by world region compared to the USA and Canada^**^			
SR	1.45 (1.22–1.72)	**2.09 (1.38**–**3.18)**	**2.43 (1.57**–**3.78)**
Combined (SR + contact aspiration)	0.99 (0.84–1.18)	**1.41 (1.10**–**1.96)**	0.94 (0.60–1.48)
Contact aspiration	**0.60 (0.46**–**0.78)**	**0.49 (0.34**–**0.70)**	**0.44 (0.27**–**0.71)**
IAT	0.96 (0.56–1.64)	0.59 (0.29–1.19)	0.97 (0.42–2.28)
No treatment	1.00 (0.49–2.02)	**0.21 (0.08**–**0.55)**	1.08 (0.47–2.47)
Other	1.11 (0.23–5.34)	0.45 (0.05–4.06)	1.24 (0.14–11.11)

*“Overall” column shows the risk ratio for preferring the row's first-line approach, for an ordinal world region outcome (from USA and Canada to Europe to the Rest of the world).*

** *USA and Canada were chosen as reference value. SR, stent-retriever; IAT, intra-arterial thrombolysis; RR, risk ratio; 95% CI, 95% confidence interval. Bold values represent statistically significant findings (p < 0.05)*.

### Influence of Availability and Access to Optimal MeVO EVT Tools

Overall, 162 (61.5%) participants felt that the current endovascular devices to treat MeVO stroke could be improved. Only 79 (30.0%) participants thought that the optimal tools already existed, and 22 (8.4%) thought that the appropriate tools currently do not exist. The interventionists who thought that the optimal tools to treat MeVO stroke do not exist were more likely to prefer stent-retrievers alone as a first-line approach [40.5%; RR 2.07, 95% CI 1.01–4.24 (ref category: tools exist)] and were more likely to treat MeVOs medically with IA tPA [12.43%; RR 3.25, 95% CI 1.71–6.12 (ref category: tools exist)].

Two-hundred-and-three (77.2%) neurointerventionists indicated that they had access to the best available tools, and 60 (22.8%) stated that they did not (always) have access to the ideal tools. Those without access to the optimal tools more frequently chose no EVT at all as a first-line approach [RR 3.41, 95% CI 1.11–10.49 (reference category: having the access to the best available tools)] (Table 3).

**Table 3:** Likelihood of preferred first-line approach (stent-retriever, combined stent-retriever and aspiration, aspiration, intra-arterial thrombolysis, or other) by physicians' opinion on whether the appropriate tools to treat MeVO exist, or whether they have access to the best available tools.

	**No (*n* = 22)**	**Room for improvement (*n* = 162)**
	**RR (95% CI)**	**RR (95% CI)**
Do appropriate tools exist for MeVOs^*^		
SR alone	**2.07 (1.01**–**4.24)**	1.11 (0.72–1.73)
Aspiration	1.56 (0.69–3.49)	1.23 (0.77–1.96)
IAT	**3.25 (1.26**–**8.42)**	0.98 (0.50–1.90)
No treatment	2.06 (0.60–7.14)	1.30 (0.55–3.10)
	**No (** ***n*** **=** **18)**	**Not in all cases (** ***n*** **=** **42)**
	**RR (95% CI)**	**RR (95% CI)**
Access to best available tools in current practice^*^		
SR alone	1.53 (0.69–3.36)	0.95 (0.58–1.55)
Aspiration	2.21 (0.93–5.29)	1.20 (0.68–2.13)
IAT	1.71 (0.64–4.57)	0.91 (0.68–2.13)
No treatment	**3.41 (1.11**–**10.49)**	0.83 (0.33–2.06)

* *“Yes” was taken as the base outcome and combined technique (stent-retriever plus contact aspiration) was chosen as reference value. Other category was included in the analysis but excluded from the table for simplicity. SR, stent-retriever; IAT, intra-arterial thrombolysis; RR, risk ratio; 95% CI, 95% confidence interval. Bold values represent statistically significant findings (p < 0.05)*.

## Discussion

This survey study found that the first-line device preferences of neurointerventionists for EVT in MeVO stroke vary based on the exact location of the occlusion, physician's region of practice, whether they think that adequate tools exist, and whether they have access to these tools in their current practice.

The optimal strategy for recanalizing MeVOs is currently not known, and the data on the efficacy of first-line aspiration vs. stent-retriever techniques are heterogenous and exclusively from non-randomized studies (12–15). Use of stent-retrievers, either alone (16–19) or combined with aspiration (10, 11), seems to be a widespread approach for MeVO EVT. Stent-retrievers alone or combined stent-retrievers and aspiration were the most commonly chosen first-line approaches, with the combined approach being preferred less often in more distal occlusions and stent-retriever alone more often in A3 occlusions.

EVT device choice in MeVO stroke is likely largely determined by the device's safety profile. Because the affected vessels in MeVO strokes are smaller, more distal, and more fragile, the risk of complications such as vasospasm, manipulation-related subarachnoid hemorrhage, and dissection is increased (3). These risks should be mitigated in order for EVT to result in a net benefit for the patients undergoing recanalization. Current large bore aspiration catheters may increase the risk of vascular damage, which is reflected in the decreased preference for first-line aspiration in more distal MCA occlusions in our study. Indeed, most intermediate catheters are 5–6Fr in diameter, and thus, they may be too large for MeVOs, considering the average diameter of the M2 middle cerebral artery segments is around 1.4–2.3 mm (20). Furthermore, when navigating the aspiration catheter to distal occlusion sites, there is the possibility of the device getting stuck at a vessel branch point, e.g., the middle cerebral artery bifurcation, although this risk can be mitigated by the use of wedge-shaped microcatheters (21), or coaxial microcatheters better size-matched to the aspiration catheter so as to reduce the step in transition. When using a primary combined approach on the other hand, limited lengths of currently available intermediate and microcatheters may render distal occlusions unreachable.

For stent-retrievers, the tortuosity and angle of the arteries may affect safety because of increased shearing at branch points during stent retrieval as well as and displacement of the arterial tree, both of which may result in subarachnoid hemorrhage. Use of stent-retrievers may hence be more desirable in straight arteries, such as the A3 where there is less tortuosity compared to the MCA branches (22).

Combined aspiration and stent-retrieval was shown to reduce the risk of distal embolization in large vessel occlusion stroke in some studies, with subsequent improved reperfusion quality and clinical outcomes (21). We found that a substantial number of physicians preferred this combined approach in the more proximal MeVO locations (M2/3 and P2/3). Recently proposed techniques such as blind exchange mini-pinning (11), in which the aspiration catheter is advanced introduced over the bare pusher wire once the stent-retriever is deployed, can circumvent problems related to catheter length and, at the same time, provide effective aspiration during the retrieval process. Techniques and specific tools for medium-sized arteries, such are mini stent-retrievers, are under development (17, 23); thus, it can be expected that more data on the safety and efficacy of these techniques will emerge soon.

Access to neurovascular tools and materials plays an important role in EVT decisions and first-line device choice for treating MeVOs as observed in this study. When practitioners thought that the appropriate tools do not exist, they more often chose to treat with stent-retrievers alone or opt for intra-arterial thrombolysis as a first-line approach. Those that felt they did not have access to the best available tools in their practice often chose not to treat at all.

Overall, interventionalists from Europe more often opted for stent-retrievers or combined stent-retrievers and aspiration as a first-line approach, whereas direct aspiration only was the more frequently preferred first-line approach in the USA and Canada. Availability of material and devices in different regions as well as local experience with these tools could potentially account for this variation in physicians' preferences as suggested in previous studies (24), in which the willingness to treat M2 occlusions increased under assumed ideal conditions in some regions. There is variability in the distribution and supply of stent retrievers across the world with some centers having access only to earlier generation devices, although device availability per country and center is hard to check and changing quickly. Physicians with no access to the optimal devices more often opted either to use stent-retrievers alone or not treat with EVT at all forgoing endovascular treatment that could potentially benefit the patient.

## Limitations

Our study has several limitations. First, decisions in endovascular treatment are highly dependent on details of patient anatomy and factors such as patient motion during EVT. Radiologic images were presented with all case scenarios to make them as realistic as possible; however, details in these images or cases may limit the generalizability of our study results to real clinical practice. Secondly, the landscape of EVT materials and tools changes fast, hence the results represent a snapshot in time and availability would differ as the tools continue to evolve in each region. In addition, the survey did not ask for the specific device brands that were available at respondents' institutions. As such, we do not know the exact EVT materials on which our results reflect, other than the devices that are currently approved in general for EVT. Our intention was to provide a general overview of the field. Thirdly, our respondent sample was collected through personal and professional networks of the study authors, which may introduce selection bias in the results (e.g., overrepresentation of teaching hospitals). Models of financial compensation for MeVO EVT may also have differed between respondents' practice settings.

## Conclusion

In this study, neurointerventionalists chose a targeted vessel-specific approach when treating MeVOs. Stent-retrievers alone or combined stent-retriever and aspiration were the most commonly used first-line approach, with the combined approach being preferred less often in more distal occlusions and stent-retriever alone more often in A3 occlusions as a first-line approach. Interventionalists from Europe used stent-retrievers and combined stent-retriever and aspiration more often as a first-line treatment, whereas direct aspiration only was more frequently preferred in North America. Physicians without access to the optimal devices more often used stent-retrievers alone or chose not to treat endovascularly at all, forgoing potential benefit.

## Data Availability Statement

The raw data supporting the conclusions of this article will be made available by the authors, without undue reservation.

## Ethics Statement

The Conjoint Health Research Ethics Board of the University of Calgary reviewed and approved this study (REB20-2086). Written informed consent to participate in this study was provided by the participants. Written informed consent was obtained from the individuals for the publication of any potentially identifiable images or data included in this article.

## Author Contributions

NK: study conception, data collection, graphical analysis, design of the work, interpretation of data, and manuscript drafting. PC, MG, and MK: study conception, data collection, design of the work, statistical analysis, interpretation of data, and manuscript drafting. JO: study conception, data collection, design of the work, interpretation of data, and manuscript editing. NSi: study conception, data collection, design of the work, statistical analysis, interpretation of data, and manuscript editing. MA: study conception, interpretation of data, and critical manuscript revision. JR: critical manuscript revision. JF and MC: study conception, data collection, design of the work, interpretation of data, and critical manuscript revision. NSa: study conception, data collection, and critical manuscript revision. RA: manuscript revisions. MH: manuscript drafting and critical manuscript revisions. All authors contributed to the article and approved the submitted version.

## Conflict of Interest

NK is an informatics consultant for Circle Neurovascular. JF reports grants and personal fees from Stryker, Acandis, Microvention, Medtronic, personal fees from Codman, Ceronovus, Penumbra, Phenox, other (stock ownership) from Tegus, outside the submitted work; and Executive functions with University Medical Center Hamburg-Eppendorf, Eppdata GmbH. MC reports personal fees from Stryker, personal fees from Microvention, Medtronic, Genentech, Ceronovus, Penumbra, outside the submitted work. MG reports personal fees from Stryker, personal fees from Mentice, personal fees from Microvention, personal fees from Medtronic, outside the submitted work; in addition, MG has a patent for Systems of acute stroke diagnosis issued and licensed. The remaining authors declare that the research was conducted in the absence of any commercial or financial relationships that could be construed as a potential conflict of interest.

## Publisher's Note

All claims expressed in this article are solely those of the authors and do not necessarily represent those of their affiliated organizations, or those of the publisher, the editors and the reviewers. Any product that may be evaluated in this article, or claim that may be made by its manufacturer, is not guaranteed or endorsed by the publisher.
