# Supplementary material for: Endovascular Device Choice and Tools for Recanalization of Medium Vessel Occlusions: Insights From the MeVO FRONTIERS International Survey
Source: Front Neurol. 2021 Sep 15;12:735899. doi: 10.3389/fneur.2021.735899 (PMC8480153; doi:10.3389/fneur.2021.735899)
Supplement: Supplementary file 1 [file Data_Sheet_1.PDF]

Supplementary Table 1: Demographics of physicians (interventionalists only) participating in the survey (n=263). *IQR, interquartile range; EVT, endovascular treatment.*

**Physician demographics**	**USA & Canada (n=73)**	**Europe (n=131)**	**Rest of the world (n=59)**
Subspecialty – *n (%)*			
Interventional Neuroradiologist	37 (50.7)	106 (80.9)	27 (45.8)
Interventional Radiologists	1 (1.4)	16 (12.2)	1 (1.7)
Interventional Neurologist	16 (21.9)	4 (3.0)	16 (27.1)
Neurosurgeon	19 (26.0)	5 (3.8)	15 (25.4)
Hospital Setting – *n (%)*			
Teaching	61 (83.6)	125 (95.4)	50 (84.8)
Non-teaching	12 (16.4)	6 (4.6)	9 (15.3)
Physician gender – *n (%)*			
Female	5 (6.9)	20 (15.3)	3 (5.1)
Male	67 (91.8)	111 (84.7)	56 (94.9)
Do not wish to declare	1 (1.4)	-	-
Age - *n (%)*			
Under 30 years	-	2 (1.5)	1 (1.7)
31 – 40 years	16 (21.9)	38 (29.0)	21 (35.6)
41 – 50 years	31 (42.5)	48 (36.6)	27 (45.8)
51 – 60 years	19 (26.3)	29 (22.1)	8 (13.6)
Over 60 years	7 (9.6)	14 (10.7)	2 (3.4)
Experience in vascular neuro-interventions – *n (%)*			
0 - 5 years	7 (9.6)	17 (13.0)	14 (23.7)
5 -10 years	23 (31.5)	26 (19.9)	13 (22.0)
10 – 15 years	14 (19.8)	38 (29.0)	16 (27.1)
15 – 20 years	12 (16.4)	25 (19.1)	9 (15.3)
More than 20 years	17 (23.3)	24 (18.3)	7 (11.9)
Range of annual center thrombectomy volume – *median*	100-200	100-200	50-100
Range of annual personal thrombectomy volume – *median*	10-50	50-100	10-50
Number of interventionalists – *median (IQR)*	4 (3-5)	4 (3-5)	2 (2-3)
Availability of 24/7 coverage for EVT – *n (%)*			
Yes	72 (98.6)	117 (89.3)	51 (86.4)
No	1 (1.4)	14 (10.7)	8 (13.6)

Supplementary Table 2. Baseline respondent characteristics and scenario occlusion location by first-line endovascular treatment device choice. Incidence rate ratios are shown from multinomial regression analysis, showing the size and significance (but not direction) of device choice preference differences between the variable categories.

	SR first	SR + aspiration	Aspiration first	IAT	No treatment	Other	IRR* (95% CI)
Occlusion location – n (%)							
M2/3	170 (32.4)	198 (37.7)	134 (25.5)	13 (2.5)	7 (1.3)	3 (0.6)	Ref.
M3	88 (33.6)	81 (30.9)	71 (27.1)	16 (6.1)	3 (1.2)	3 (1.2)	**1.09 (1.00-1.20)**
M3/4	73 (27.9)	40 (15.3)	68 (26.0)	50 (19.1)	29 (11.1)	2 (0.8)	**1.65 (1.48-1.84)**
A3	207 (39.4)	131 (25.0)	149 (28.4)	25 (4.8)	10 (1.9)	3 (0.6)	1.02 (0.92-1.13)
P2/3	72 (27.5)	97 (37.0)	70 (26.7)	15 (5.7)	5 (1.9)	3 (1.2)	**1.16 (1.05-1.29)**
Respondent age (range) – n (%)							
<30 years	6 (35.3)	7 (41.2)	3 (17.7)	0	1 (5.9)	0	Ref.
31-40 years	157 (29.9)	168 (32.0)	140 (26.7)	38 (7.2)	17 (3.2)	5 (1.0)	1.26 (0.61-2.60)
41-50 years	261 (35.2)	225 (30.3)	191 (25.7)	39 (5.3)	24 (3.2)	2 (0.3)	1.12 (0.54-2.31)
51-60 years	133 (34.0)	101 (25.8)	109 (27.9)	33 (8.4)	11 (2.8)	4 (1.0)	1.24 (0.60-2.60)
>60 years	53 (32.9)	46 (28.6)	49 (30.4)	9 (5.6)	1 (0.6)	3 (1.9)	1.20 (0.56-2.59)
Respondent sex – n (%)							
male	541 (33.1)	477 (29.1)	452 (27.6)	107 (6.5)	52 (3.2)	8 (0.5)	Ref.
female	69 (35.9)	64 (33.3)	40 (20.8)	11 (5.7)	2 (1.0)	6 (3.1)	0.96 (0.69-1.35)
Neuro-interventional experience (range) – n (%)							
0-5 years	95 (32.3)	81 (30.9)	66 (25.2)	16 (6.1)	4 (1.5)	0	Ref.
5-10 years	121 (27.9)	142 (32.7)	120 (27.7)	30 (6.9)	16 (3.7)	5 (1.2)	1.23 (0.93-1.64)
10-15 years	156 (32.8)	136 (28.6)	135 (28.4)	35 (7.4)	13 (2.7)	1 (0.2)	1.13 (0.86-1.49)
15-20 years	136 (42.2)	95 (29.5)	58 (18.0)	16 (5.0)	16 (5.0)	1 (0.3)	0.97 (0.70-1.33)
>20 years	102 (30.5)	91 (27.2)	108 (32.2)	22 (6.6)	5 (1.5)	7 (2.1)	1.23 (0.90-1.68)
Career stage – n (%)							
In training	22 (30.1)	32 (43.8)	17 (23.3)	1 (1.4)	1 (1.4)	0	Ref.
Board certified <5 years	75 (34.6)	65 (30.0)	56 (25.8)	17 (7.8)	4 (1.8)	0	1.12 (0.74-1.71)
Board certified 5-10 years	113 (26.9)	132 (31.4)	142 (33.8)	12 (2.9)	16 (3.8)	5 (1.2)	1.3 (0.88-1.92)
Board certified >10 years	400 (35.5)	318 (28.2)	277 (24.6)	89 (7.9)	33 (2.9)	9 (0.8)	1.18 (0.81-1.70)
Center yearly EVT volume (range) – n (%)							
<50	79 (37.6)	46 (21.9)	61 (29.1)	21 (10.0)	2 (1.0)	1 (0.5)	Ref.
50-100	135 (34.9)	128 (33.1)	96 (24.8)	9 (2.3)	19 (4.9)	0	0.94 (0.69-1.27)
100-200	213 (29.3)	232 (31.9)	207 (28.4)	41 (5.6)	23 (3.2)	12 (1.7)	1.10 (0.84-1.43)
>200	183 (35.8)	141 (27.6)	128 (25.1)	48 (9.4)	10 (2.0)	1 (0.2)	0.98 (0.74-1.31)
Personal yearly EVT volume (range) – n (%)							

<10	17 (22.1)	30 (39.0)	16 (20.8)	12 (15.6)	2 (2.6)	0	Ref.
10-50	317 (32.9)	250 (25.9)	288 (29.8)	65 (6.7)	33 (3.4)	12 (1.2)	0.92 (0.69-1.23)
50-100	216 (34.3)	205 (32.5)	151 (24.0)	38 (6.0)	18 (2.9)	2 (0.3)	0.81 (0.61-1.09)
>100	35 (27.8)	57 (45.2)	29 (23.0)	4 (3.2)	1 (0.8)	0	0.76 (0.53-1.08)
World region – n (%)							
USA and Canada	91 (17.8)	128 (25.1)	221 (43.3)	42 (8.2)	24 (4.7)	5 (1.0)	Ref.
Europe	340 (37.3)	322 (35.3)	193 (21.2)	44 (4.8)	9 (1.0)	4 (0.4)	**0.61 (0.52-0.72)**
Rest of the world	179 (43.3)	97 (23.5)	78 (18.9)	33 (8.0)	21 (5.1)	5 (1.2)	**0.70 (0.55-0.89)**

Supplementary Methods

MeVO-FRONTIERS

All case-scenarios were composed of fictional information as well as individual values of multiple patients were combined. As such, all presented data are fictional, not traceable to any patient, and completely anonymous.

Case 1/7

1.1image Multiphase CTA with right MeVO (medium vessel occlusion) - M2/M3 MCA (pink arrowheads). Delayed washout in the third phase (III).

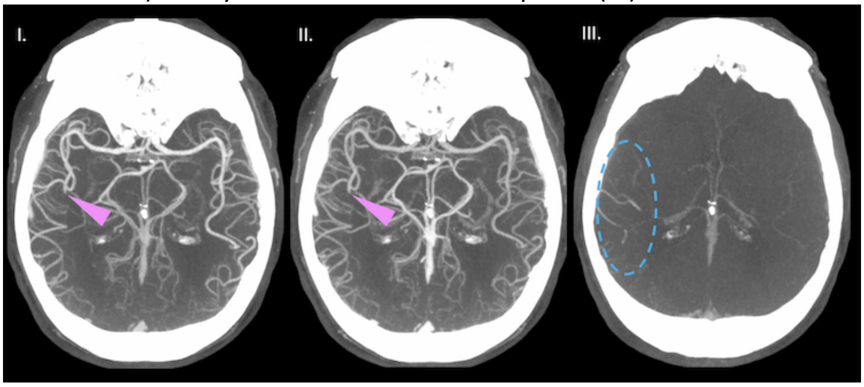

1.2image Sagittal and coronal view: Pink arrowheads pointing to MeVO, red line represents course of missing artery.
1.3image CT perfusion – Tmax map.

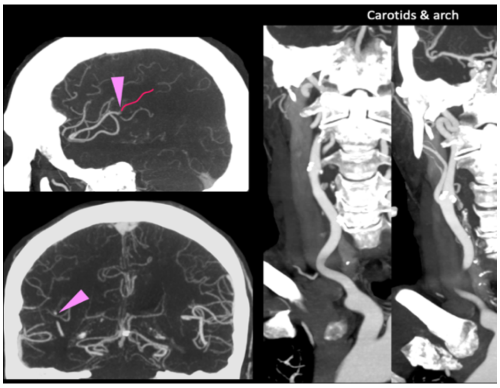

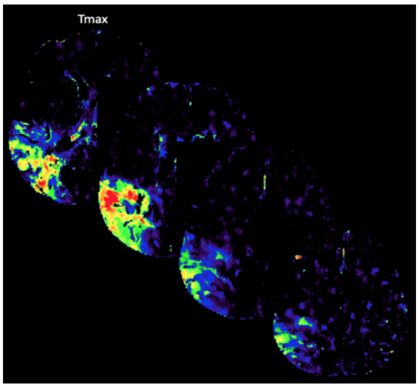

Q1 If you were to treat this MeVO, what would be your preferred **first-line approach**?

- ◯ Direct contact aspiration
- ◯ Stent-retriever
- ◯ Combined stent-retriever and contact aspiration
- ◯ Intra-arterial thrombolytics
- ◯ Other (specify) ____
- ◯ Not applicable (I am not an interventionist)
- ◯ I would not treat any of the presented scenarios

Case 2/7
Q2.1image MRA and DSA images demonstrating right terminal ICA occlusion.

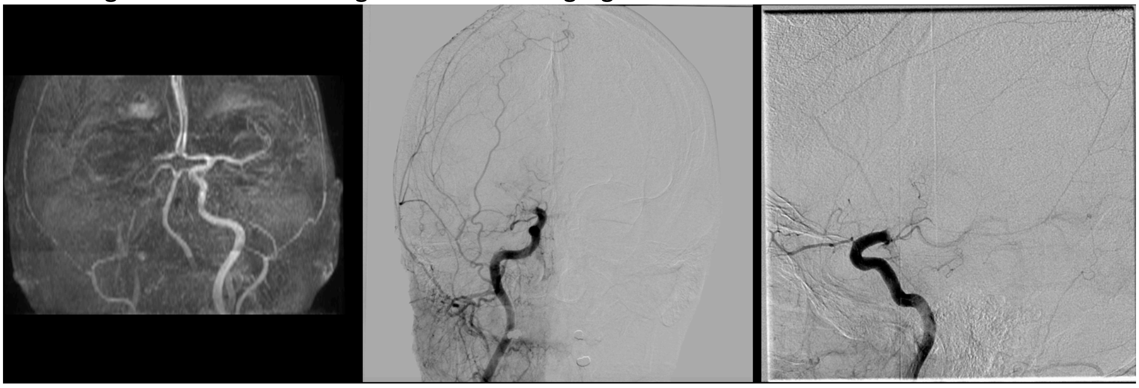

Q2.2image After the first stent-retriever pass, there is TICI 2C reperfusion in the MCA territory, *but* embolisation into the **right A3 ACA (secondary MeVO).** The red line represents the course of the missing artery.

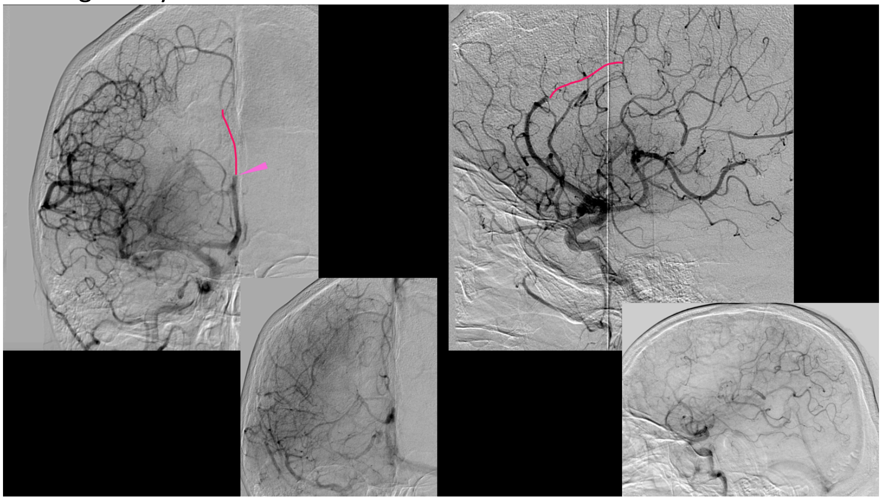

Q2 If you were to treat this MeVO, what would be your preferred **first-line approach**?

- ◯ Direct contact aspiration
- ◯ Stent-retriever
- ◯ Combined stent-retriever and contact aspiration
- ◯ Intra-arterial thrombolytics
- ◯ Other (specify) ____
- ◯ Not applicable (I am not an interventionist)
- ◯ I would not treat any of the presented scenarios

Case 3/7

Q3.1image Right MeVO - A3 ACA (pink arrowhead). Red line on the sagittal projection represents the course of the missing artery. Turquoise line represents the course of the terminal ICA and the ACA origin.

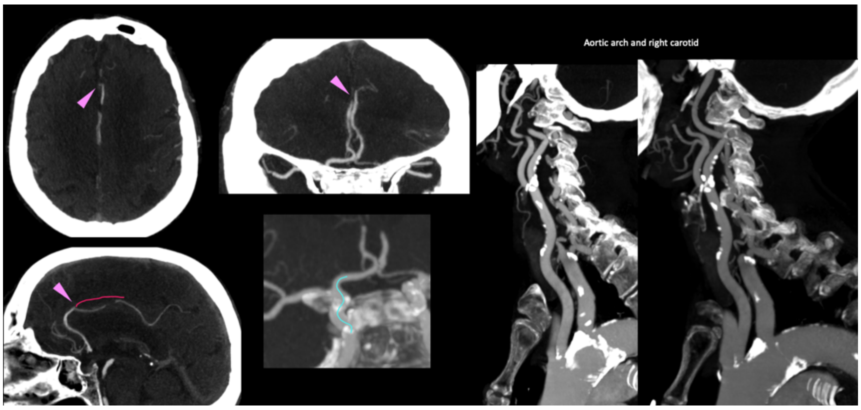

Q3.2image

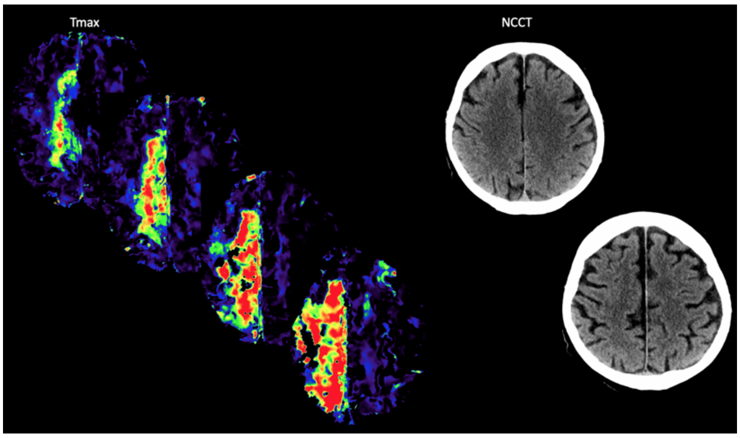

Q3 If you were to treat this MeVO, what would be your preferred **first-line approach**?

- ◯ Direct contact aspiration
- ◯ Stent-retriever
- ◯ Combined stent-retriever and contact aspiration
- ◯ Intra-arterial thrombolytics
- ◯ Other (specify) ____
- ◯ Not applicable (I am not an interventionist)
- ◯ I would not treat any of the presented scenarios

Q4 **Case 4/7**

Q4.1image First DSA run demonstrating left middle M1 MCA occlusion.

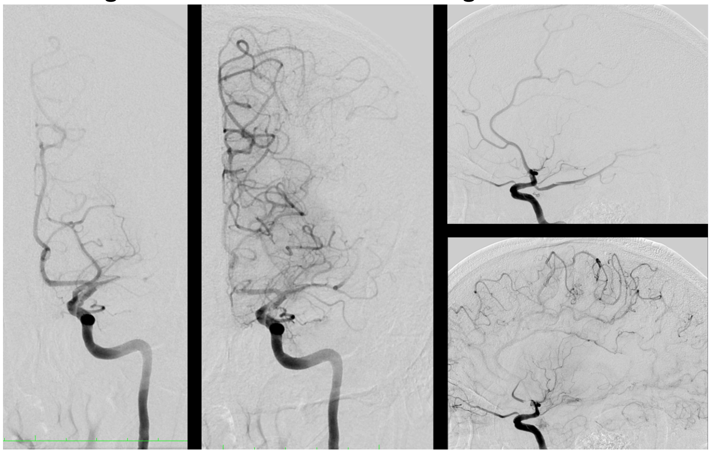

Q4.2image After the first stent-retriever pass, there is TICI 2B reperfusion, *but* embolisation into an **M3/M4 branch (secondary MeVO).** The red line represents the course of the missing artery.

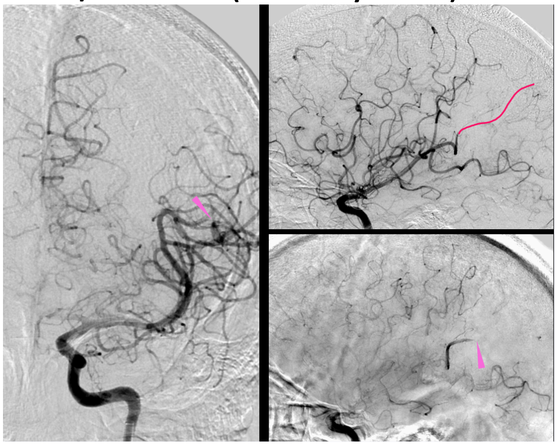

Q4 If you were to treat this MeVO, what would be your preferred **first-line approach**?

- ◯ Direct contact aspiration
- ◯ Stent-retriever
- ◯ Combined stent-retriever and contact aspiration
- ◯ Intra-arterial thrombolytics
- ◯ Other (specify) ____
- ◯ Not applicable (I am not an interventionist)
- ◯ I would not treat any of the presented scenarios

Q5 Case 5/7

Q3.1image Multiphase CTA with left MeVO - M3 MCA (pink arrowheads). Delayed washout in the third phase (III).

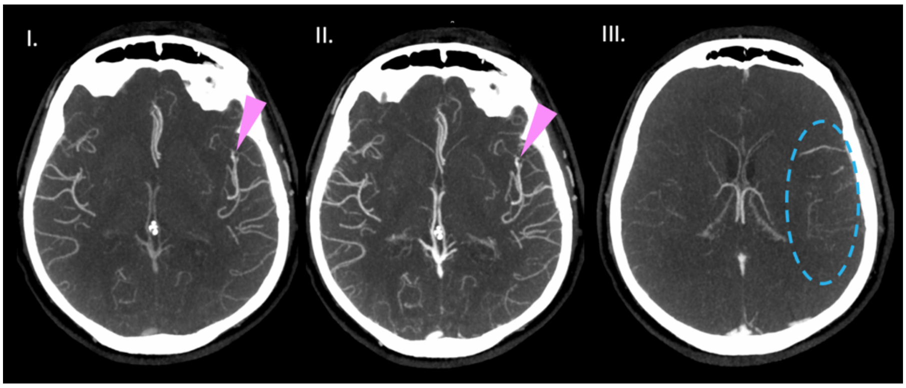

Q3.2image Sagittal and coronal view: Pink arrowheads pointing to MeVO, red line represents course of missing artery.

Q3.3image CT perfusion – Tmax map

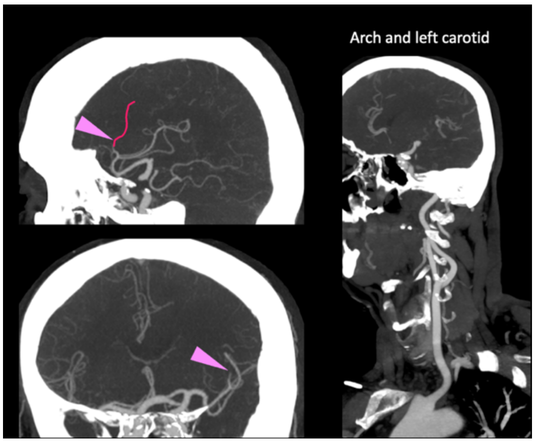

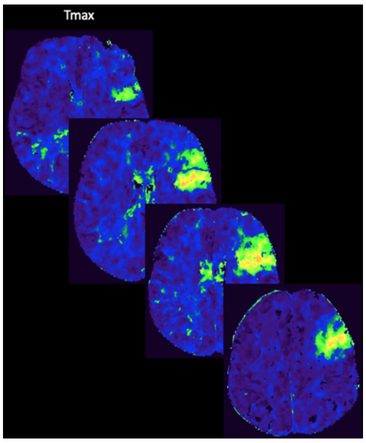

Q5 If you were to treat this MeVO, what would be your preferred **first-line approach**?

- ○ Direct contact aspiration
- ○ Stent-retriever
- ○ Combined stent-retriever and contact aspiration
- ○ Intra-arterial thrombolytics
- ○ Other (specify) ____
- ○ Not applicable (I am not an interventionist)
- ○ I would not treat any of the presented scenarios

Case 6/7

Q6.1image Multiphase CTA shows a right mid-M1 MCA occlusion and delayed washout in the third phase (III). Coronal CTA MIP reconstruction shows the course of the right CCA and ICA.

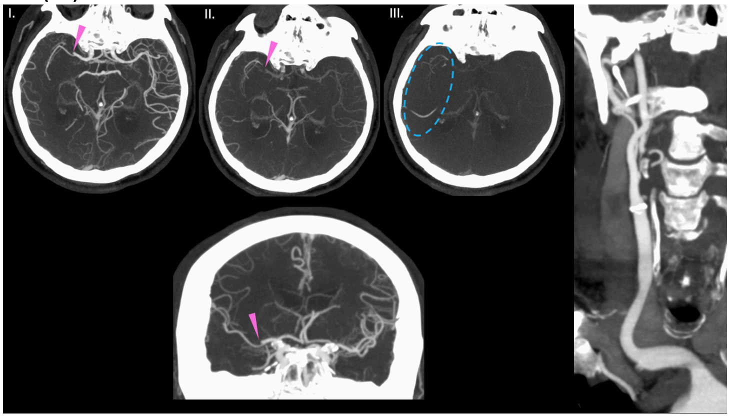

Q6.2image First intracranial DSA run shows recanalization of the right M1 MCA and new **secondary MeVO in M2/M3** superior MCA branch.

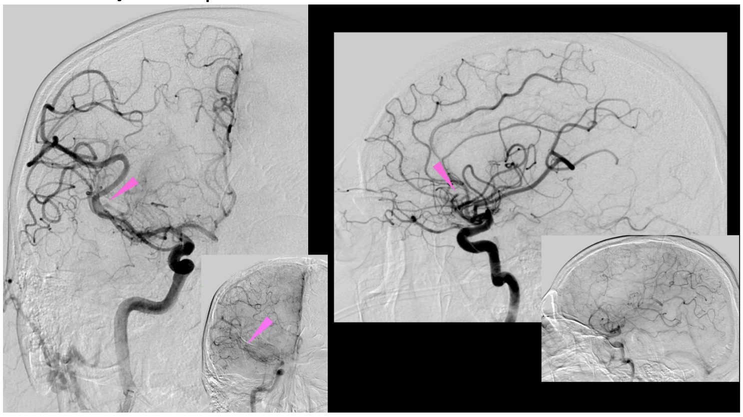

Q6 If you were to treat this MeVO, what would be your preferred **first-line approach**?

- ◯ Direct contact aspiration
- ◯ Stent-retriever
- ◯ Combined stent-retriever and contact aspiration
- ◯ Intra-arterial thrombolytics
- ◯ Other (specify) ____
- ◯ Not applicable (I am not an interventionist)
- ◯ I would not treat any of the presented scenarios

Q7 Case 7/7

Q7.1image CTA shows a right MeVO - P2/P3 PCA.

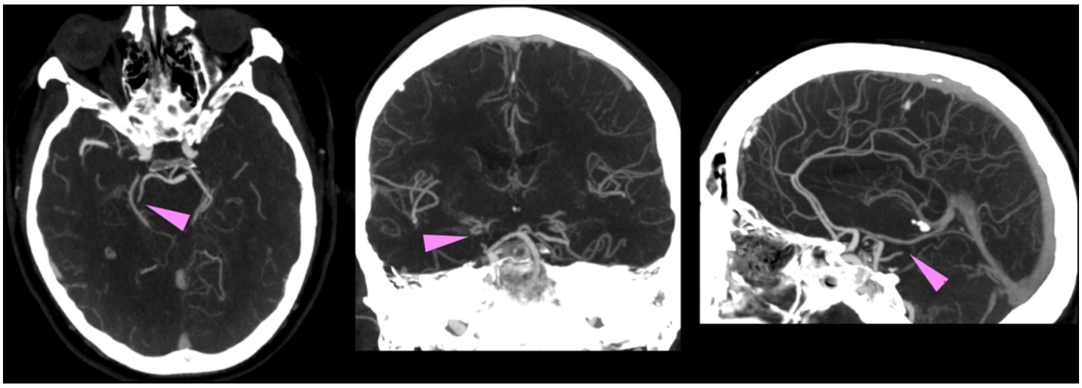

Q7.2image Coronal CTA MIP shows the access pathway through the right vertebral artery, the origin of the right and left vertebral artery and the aortic arch.

Q7.3image CT perfusion – Tmax map

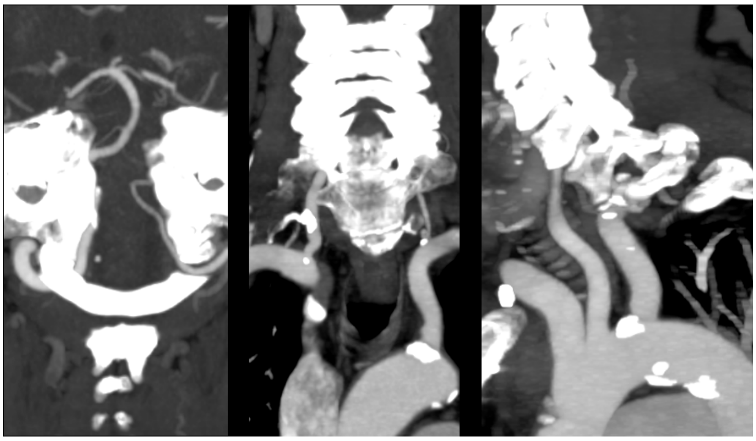

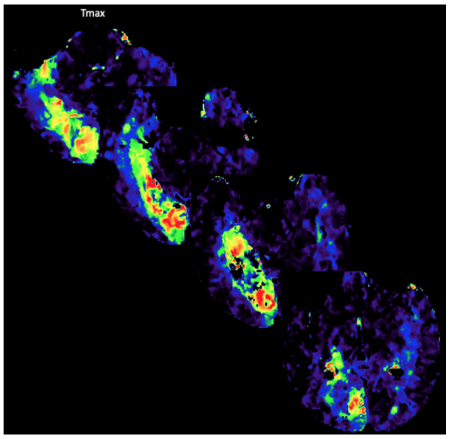

Q7 If you were to treat this MeVO, what would be your preferred **first-line approach**?

- ◯ Direct contact aspiration
- ◯ Stent-retriever
- ◯ Combined stent-retriever and contact aspiration
- ◯ Intra-arterial thrombolytics
- ◯ Other (specify) ____
- ◯ Not applicable (I am not an interventionist)
- ◯ I would not treat any of the presented scenarios

Q8.1 Do you think that **appropriate tools/devices exist** to treat the presented MeVOs?

- ◯ Yes (1)
- ◯ No (2)
- ◯ Yes, but there is substantial scope for improvement/further development (3)

Q8.2 Do you have access to the currently **best available tools/devices at your institution** to treat the presented MeVOs?

- ◯ Yes (1)
- ◯ No (2)
- ◯ Not in all cases (3)
